# Late-Onset Sacroiliac Osteoarthritis After Surgical Symphysiotomy: A Case Report

**DOI:** 10.7759/cureus.11769

**Published:** 2020-11-29

**Authors:** Sujit K Tripathy, Sudeep K Samanta, Paulson Varghese, Saurav N Nanda, Kanhaiyalal Agrawal

**Affiliations:** 1 Orthopaedics, All India Institute of Medical Sciences, Bhubaneswar, IND; 2 Orthopaedics, Kalinga Institute of Medical Sciences, Bhubaneswar, IND; 3 Nuclear Medicine, All India Institute of Medical Sciences, Bhubaneswar, IND

**Keywords:** symphysis pubis, sacroiliac joint, pregnancy, childbirth, postpartum, pubic diastasis

## Abstract

A 35-year-old female presented with right-sided gluteal pain and difficulty in walking 10 years after surgical symphysiotomy. Radiograph of the pelvis and bilateral hip joints showed osteoarthritis of the right sacroiliac joint with pubic diastasis of 1.5 cm. She was operated with pubis symphysis reduction and fixation using two orthogonal plates with one iliosacral screw. Postoperative period was uneventful. She was able to walk independently after three months of fixation. Follow-up at 18 months showed complete relief of symptoms with maintenance of reduction and no hardware breakage. The Lindahl score was 78, indicating an excellent outcome.

## Introduction

Symphysiotomy is performed during attempted vaginal delivery by dividing the fibrocartilagenous joint of the symphysis pubis. Though rarely required in current obstetric practice, it can be a life-saving procedure in remote, resource-limited settings [1]. Physiological changes during pregnancy and labour cause significant relaxation of supporting ligaments and other connective tissue of the pelvis. This leads to an increase in pelvic diameter facilitating childbirth. The degree to which these hormonally mediated changes happen varies considerably [2,3]. In some cases, diastasis of pubic symphysis can occur with separation of pubic rami greater than 1 cm, and it is then considered as pathological pubic diastasis [4]. A higher degree of symphyseal separation with symphyseal diastasis >2.5 cm indicates injury to the posterior pelvic arch, including the sacroiliac joint [5-7].

The resulting pelvic instability from symphysiotomy may lead to significant morbidity in the postpartum period. The immediate postpartum period may be complicated by symphyseal hematoma, osteitis pubis, vesicovaginal or vesicocutaneous fistulae, stress incontinence, and pain on walking. The usual treatment is conservative. Surgical treatment may be indicated when conservative measures fail; however the research of this is limited to a few case reports in the literature [4,5,8-11]. The management of chronic cases is more challenging as these patients develop parasymphyseal degeneration and sacroiliac arthritis after many years of symphysiotomy [12]. Naijobi et al. recommended fusion of the symphysis pubis in chronic cases of more than six months duration if it is symptomatic [4]. We report the surgical fixation of the pelvic ring in a 35-year-old female who developed sacroiliac joint arthritis 10 years after surgical symphysiotomy.

## Case presentation

A 35-year-old female presented to us with right-sided gluteal pain and difficulty in standing and walking. The symptoms started very slowly following her vaginal delivery 10 years back; she had a history of surgical symphysiotomy for obstructed labour. The patient recalls mild pain over the pubic area and low back in the initial postpartum period. The obstetrician advised her a pelvic binder and analgesic for six weeks. She had persistent mild pain after six weeks, but she could manage with small doses of analgesic (tablet paracetamol 650mg on demand). However, her pain intensity increased significantly in the gluteal region for the last three years. It was managed by a local doctor with analgesics and pelvic binder. Gradually the pain increased and became severe enough (visual analogue scale of 8/10) to restrict her daily activities. She was not able to walk at a stretch beyond a distance of around 20 metres. However, she had no history of recent trauma, fever or other joint symptoms. On examination, she had severe tenderness over the right sacroiliac joint and gluteal region with no radicular pain to the lower limb. She had an antalgic gait, and was not able to perform one-leg standing on the right side. Pelvic compression and distraction tests, FABER (Flexion, Abduction and External Rotationtest) test, and Gaenslen's test were positive, indicating pathology in the sacroiliac (SI) joint. The pubic symphysis region was also tender on palpation. There was no neurological deficit, and both hip joints were normal on examination.

**Figure 1:**
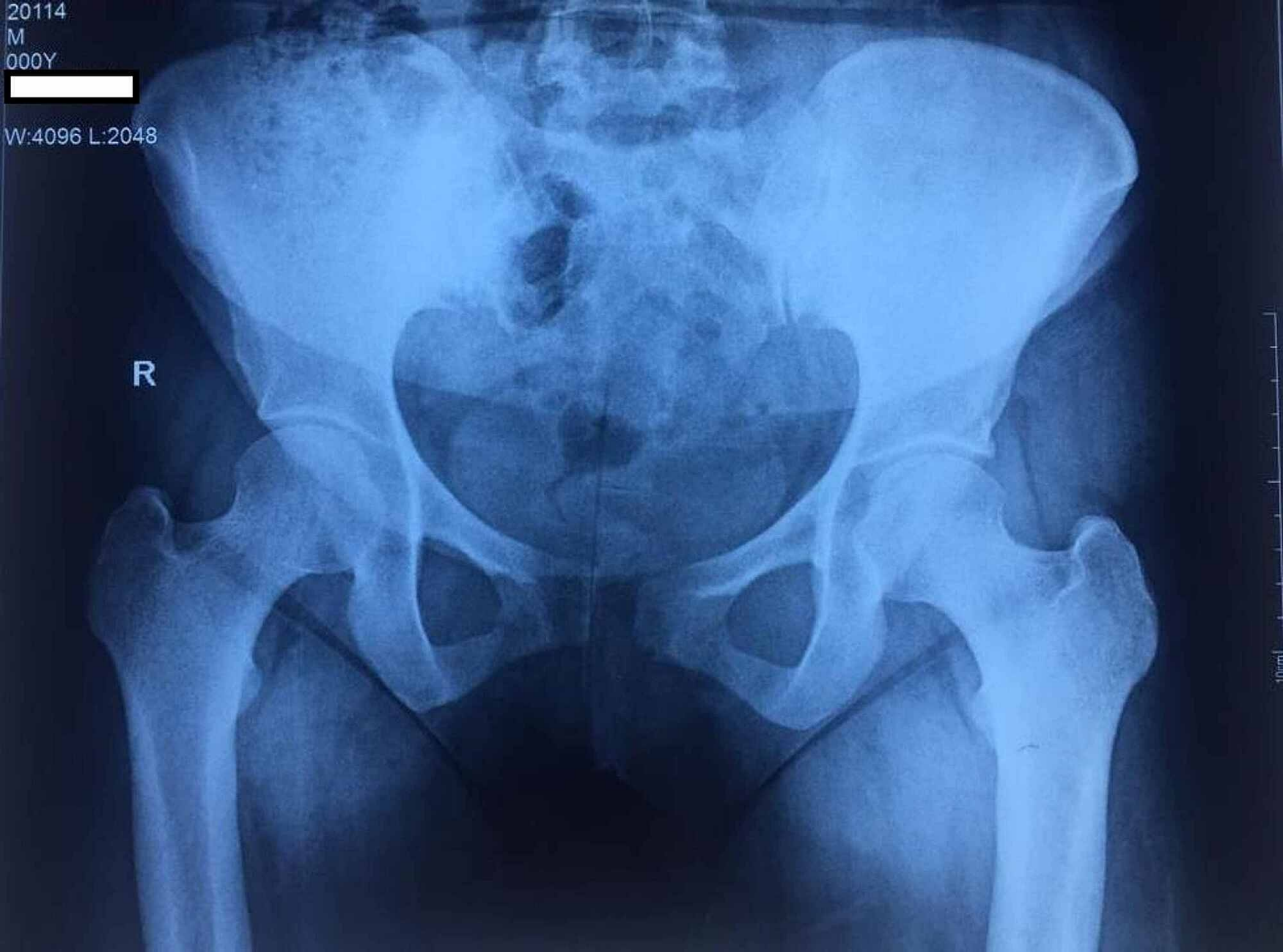
Anteroposterior radiograph of pelvis with both hip joints showed sacroiliac arthritis and symphysis pubis widening of 15 mm

On standard pelvic x-ray pubic symphysis separation of 15 mm with minimal vertical displacement was noted. There were arthritic changes in the right-sided sacroiliac joint (Figure 1).

**Figure 2:**
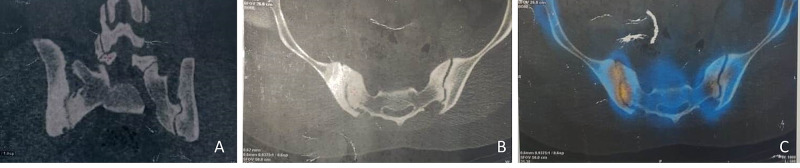
Computed tomographic scan (A, B) and the nuclear scan showed sacroiliac arthritis and increased uptake in the right sacroiliac joint

The computed tomographic scan and three-phase bone scan showed arthritic changes over the right sacroiliac joint (Kellgren-Lawrence grade 3) with subchondral sclerosis (Figure 2). Magnetic resonance imaging was also indicative of degenerative changes in the sacroiliac joint with no effusion. Haematological parameters were within the normal limit (erythrocyte sedimentation rate (ESR) 22, normal C-reactive protein level). The patient was evaluated for inflammatory disorders (rheumatoid (RA) factor, human leukocyte antigen B27 (HLA-B27), antinuclear antibody (ANA)), but found to be normal. 

**Figure 3:**
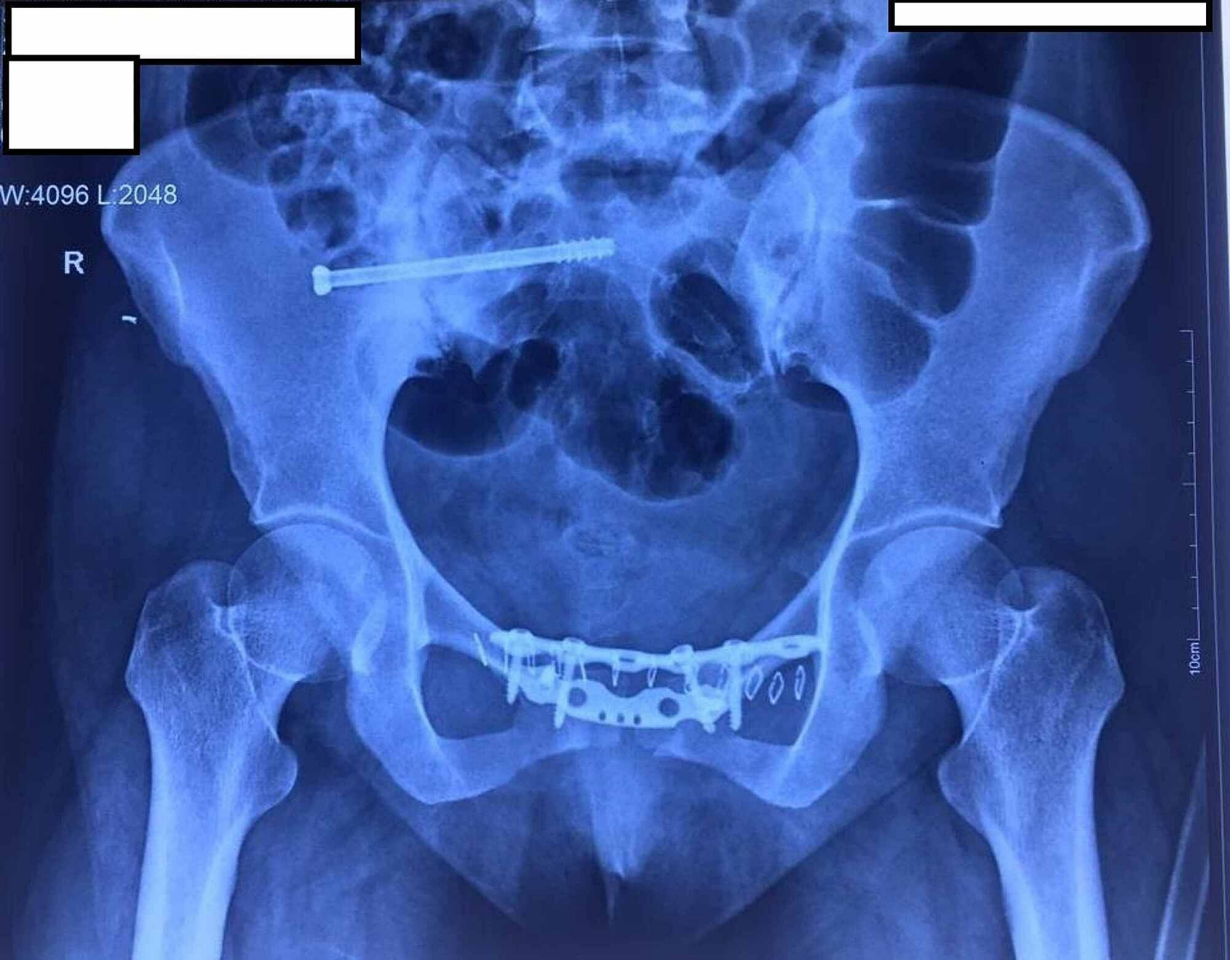
Postoperative radiograph showed well-reduced symphysis pubis (width 3 mm) with fixation using two symphyseal plates (3.5 mm reconstruction plate) placed in the orthogonal plane along with one iliosacral screw

Because of chronic injury with arthritic changes, the fusion of symphysis pubis and sacroiliac joints was recommended. However, she did not consent for the fusion and requested for an attempt of fixation. Hence, open reduction of symphysis pubis with internal fixations of both anterior and posterior segment was planned. The patient was placed supine on a radiolucent table. By Pfannenstiel approach, exposure of pubic symphysis with bilateral superior pubic rami was done. The interposed fibrous tissue between the articular parts of symphysis pubis was excised. The symphysis pubis was reduced with a pointed reduction clamp. Then internal fixation was done with two 3.5 mm reconstruction plates placed over the superior part and anterior part. After that, stabilisation of the right sacroiliac joint was done with a 7 mm partially threaded cancellous screw percutaneously (Figure 3).

**Figure 4:**
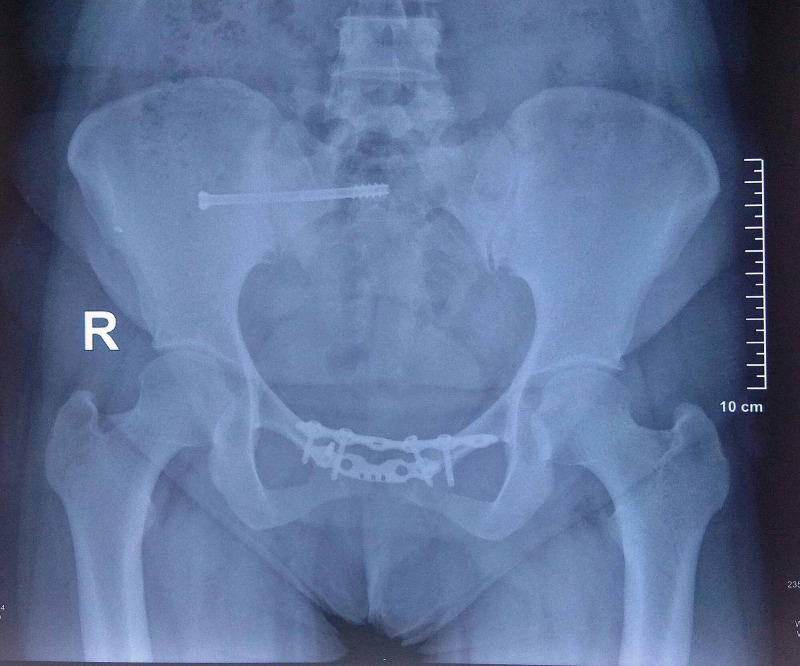
Follow up radiograph at one year showed well-maintained reduction with intact plates and screws

**Figure 5:**
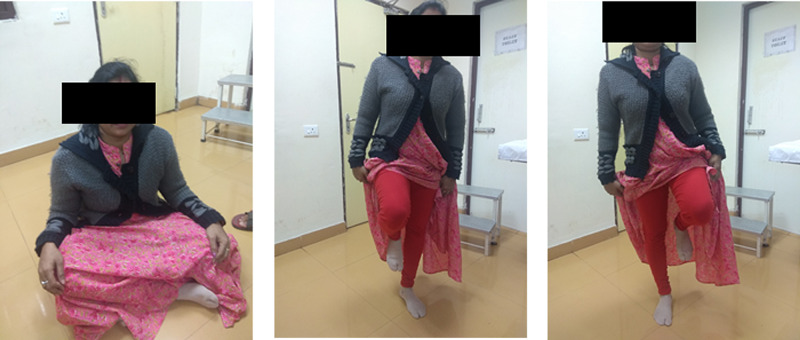
Functional ability of the patients after 18 months (A, able to sit cross-legged, B and C; able to stand on each leg independently)

The postoperative course was uneventful. Gradual assisted weight-bearing with a walker was started as per tolerance. She started walking independently after three months. Subsequently, she was followed up after six, 12, and 18 months. There was no pain in the gluteal region or sacroiliac joint after 18 months. The x-ray showed a stable pelvis with no loss of reduction at most recent follow-up (Figure 4). She was able to perform daily activities, including cross leg sitting and one-leg standing without pain (Figure 5). The Lindahl score was 78, indicating an excellent outcome. She had no dyspareunia or other implant-related complications.

## Discussion

Symphyseal and sacroiliac fixations for symptomatic sacroiliac arthritis in a young female were shown to have excellent short term outcomes in this case report. There are very few studies on long term orthopaedic complications due to instability of sacroiliac joint following symphysiotomy during childbirth [12]. 

A case-control study by Galbraith et al. showed that 80% of patients had high-grade SI joint arthritis following symphysiotomy after mean follow-up of 41.6 years [12]. The incidence of radiographic SI joint arthritis was only 16% in the control group. The patients with a higher grade of SI joint arthritis correlated with advanced age, wider separation and vertical translation of pubic symphysis. Multiparity, advanced age and obesity are major risk factors for sacroiliac joint osteoarthritis [12,13]. Biomechanically there is enough evidence that symphyseal resection exerts more rotational force on the sacroiliac joint causing arthritis changes [14]. Hence, wider separation and more vertical translation causing higher grade SI arthritis are understandable. However, in this report, the female was primigravida, and she had symphyseal widening of 14 mm only with the vertical translation of 3 mm. She developed severe symptomatic SI arthritis 10 years after surgical symphysiotomy. Probably she was inadequately treated initially without the supervision of an orthopedic surgeon.

Several reports have mentioned that the majority of spontaneous pubic ruptures do well with conservative treatment [1,2,15,16]. Bed rest, pelvic binder, special braces and local steroid injection provides good symptomatic relief in the majority of cases in acute settings. The threshold for surgical intervention in acute and subacute cases is controversial. Naijobi et al. and Haegen recommended surgery in all symptomatic patients with a symphyseal widening of more than 10 mm and a vertical translation of more than 5 mm [4,17]. Kharrazi et al. reported poor outcome with conservative treatment in severe pelvic dislocations. They recommended a formal examination of symphyseal and sacroiliac instability under anaesthesia, followed by anterior plate fixation for patients with >4 cm symphyseal widening [6]. We believe that a trial of conservative treatment must be given to all patients with acute and subacute presentations. Nevertheless, the guideline for the management of chronic symptomatic cases is operative only [4].

Naijobi et al. treated three chronic symphysiotomy patients with symphysis fusion using the plate and bone graft [4]. Rommens et al. described good results in two subacute and one chronic case with open reduction and internal fixation of the symphysis [5]. The major controversy in chronic cases is whether to fuse or fix. Few authors believe that the impaired healing potential of interposed fibrous tissue in chronic cases with disuse osteoporosis causing poor purchase of screws over the rami favours fusion [4]. However, the success of healing even after fixations in few studies prompts surgeons to attempt for fixation in selective cases where bone quality is good [5]. The reason for an excellent outcome in the present case is rigid fixation using two orthogonal plates in the symphyseal region and one iliosacral screw. This allowed adequate healing in the symphyseal region and sacroiliac joint. After 1.5 years follow up there was no loss of reduction or osteolysis around screws indicating ligament healing was strong enough to withhold any shear or distractive force along the SI joint and the symphysis pubis.

## Conclusions

In conclusion, chronic symphysiotomy may present with isolated sacroiliac arthritic features without symphysis problem. Even an asymptomatic pubic diastasis of <2.5 cm may develop sacroiliac instability and arthritis on a longer run. Close follow up of such patients for a longer duration may be warranted, and if needed, an early surgical fixation should be performed to reduce morbidity. Rigid surgical fixation of the symphysis pubis and sacroiliac joint in chronic cases may provide good relief of symptoms.
